# Challenges and Actions of IAQ under COVID-19: A Survey of Taiwanese People’s Perception of Epidemic Prevention and Indoor Places Certification

**DOI:** 10.3390/ijerph192214942

**Published:** 2022-11-13

**Authors:** Chih-Pei Hu, Jen-Hsiung Cheng

**Affiliations:** 1 Department of Public Administration, Chung Hua University, Hsinchu 30012, Taiwan; 2 Taiwan Indoor Environment Quality Management Association, New Taipei City 23555, Taiwan

**Keywords:** IAQ, COVID-19 prevention, clean and safe certification, Taiwan survey

## Abstract

COVID-19 is still spreading around the world, and the pandemic has awakened the public’s attention to environmental cleanliness. This article used an online survey for people living in Taiwan, and a total of 1206 valid questionnaires were collected in October 2021. According to the survey results of Taiwanese people’s awareness of and needs for epidemic prevention and IAQ, 94.4% of the respondents agreed that maintaining IAQ during the COVID-19 pandemic is very important for prevention. In addition, 95.4% of them also pointed out that the “Clean and Safe” mark certification should be promoted in public places. Finally, this article also uses hierarchical regression to analyze public perceptions of seven indoor places, including elevators, restaurants, dwellings, offices, gyms, kindergartens, and long-term care centers. The results found that: (1) from the perspective of epidemic prevention, improving IAQ through ventilation strategies could prevent the spread of the COVID-19 pandemic, and (2) from the perspective of promotion certification, the elevators, restaurants and offices could establish strengthened IAQ, dwellings, gyms and long-term care centers should emphasize the display of IAQ information in entrances and exits, and kindergartens should focus on increasing safety and reducing infection.

## 1. Introduction

COVID-19 is still spreading around the world, and the pandemic has awakened the public’s attention to environmental cleanliness. According to the guidance issued by the Centers for Disease Control and Prevention in United State (US CDC) on 7 May 2021, COVID-19 may be spread by inhalation of the virus in air more than six feet away from the source of infection [1]. After this, the Environmental Protection Agency in United States (US EPA) also announced the “Clean Air in Buildings Challenge“ on 17 March 2022 [2], and this is an action to help building owners and operators reduce indoor risks from airborne viruses and other pollutants. By improving ventilation and IAQ, it helps to better protect the health of building occupants and reduce the risk of spreading COVID-19.

Indoor air quality (IAQ) refers to the air quality in and around buildings and structures, in particular it relates to the health and comfort of building occupants [3]. Traditionally, IAQ has been concerned with various indoor and outdoor pollutants, as well as the impact on people indoors. In fact, people spend more than 90% of their time in an indoor environment, and many indoor spaces are densely populated but do not provide enough fresh air [4], which increases the risk of airborne infection [5]. However, with the spread of the COVID-19 pandemic, the focus of discussing IAQ in recent years has begun to shift to improving air quality and preventing the spread of the pandemic.

Therefore, the main purpose of this article is to explore the subjective evaluation of respondents to IAQ during the COVID-19 pandemic, and how to improve it. In addition, it also refers to the practices of many countries and international organizations that began to voluntarily promote the certification of air quality marks to provide a safe and clean indoor environment, so that people can remain in an area and breathe confidently. This article uses an online survey method executed in October 2021 to analyze the opinions and views of respondents living in Taiwan.

### 1.1. IAQ under COVID-19

#### 1.1.1. Strategies to Improve IAQ

Improving IAQ has already become a global phenomenon, involving every citizen, whether they are workers or not, and every country where they live, work, or attend schools, hospitals, and use transport facilities [6]. Results of the rapid spread of COVID-19 reveal the long-term underestimated impact of IAQ on human health. According to the related research, [7,8] proved that using IAQ appropriate control strategies can reduce airborne infection risk from COVID-19, and these strategies have been recommended by the World Health Organization (WHO) [9], US CDC [10] and the American Society of Heating, Refrigerating and Air-Conditioning Engineers (ASHRAE) [11]. Some strategies focus primarily on reducing airborne transmission, including increasing ventilation rates, upgrading air filters, using air purifiers or building the ultraviolet (UV) sterilization systems in rooms, and advising people to wear masks. At the same times, air purification technologies have also been introduced to mitigate the spread of diseases through aerosols.

Awada et al. [12] pointed out that professionals will pay more attention to ventilation systems to maintain high indoor quality and limit infection in indoor spaces after the pandemic. Therefore, IAQ is proposed as a fundamental way to prevent the spread of airborne viruses and maintain low levels of pollutants in indoor environments [13]. Lovec et al. [14] also argued that numerous studies in recent years have proven that better IAQ can reduce the risk of the indoor spread of the coronavirus. In particular, manual ventilation was often used to provide fresh air during the pandemic, regardless of outdoor conditions and heat loss. Insufficient indoor ventilation will lead to increased concentrations of pollutants that carry the coronavirus, and this finding increased interest in IAQ research, and analysis of the various ways to mitigate and prevent the spread of coronavirus to achieve high air quality [15].

As Abouleish [16] mentioned, in the face of uncertainty about the risk of contracting COVID-19, people’s perceptions play an important role in perceiving the uncertain risk, and these perceptions greatly influenced their actions. Actually, there are many ways to maintain IAQ. D’Antonio [17] argued that limiting the time spent inside buildings, physically removing sources of contamination, cleaning surfaces, improving ventilation, filtering the air, and cleaning the air can assist in maintaining the IAQ. Generally, current IAQ control strategies can be broadly divided into three categories and integrating them correctly will provide the most effective performance against the coronavirus [7]. (see Table 1).

1. Source control: this is the first and most important IAQ control strategy. For the COVID-19 pandemic, this means testing, tracking and isolating infected people, and preventing the spread of asymptomatic carriers of the virus. Local exhaust is another effective source control strategy. It has the potential to improve air quality and reduce cross-contamination by a factor of 1.4 to 10, depending on the local zoning configuration, exhaust location and flow rate [18].

2. Ventilation: ventilation refers to the supply and delivery of sufficient clean outdoor air to the breathing zone and effective dilution of contaminant concentrations. A ventilation strategy must be implemented to distribute good indoor air evenly, deliver fresh air to the breathing zone, avoid indoor cross-contamination, and remove contaminants.

3. Air cleaning: Air cleaning strategies include filtration or purification of outdoor air, recycling it into a room or a specific indoor space, and application at the individual level (e.g., wearing masks properly).

#### 1.1.2. Strengthening Ventilation Is a Key Factor in Preventing Epidemics

Research on IAQ, from the 19th century miasma theory to the 20th century correlations with sick building syndrome (SBS), has recently focused on enhancing ventilation to reduce the concentration of specific pollutants in the air. Nafchi et al. [19] pointed out that a ventilation system’s design and operation are major factors affecting aerosol transport in buildings, and the COVID-19 pandemic has created distrust in building ventilation and design systems [20].

By reviewing the recent empirical studies on IAQ and COVID-19, the following characteristics can be summarized (see Table 2):

1. Research has mainly focused on schools and classrooms: during the COVID-19 pandemic, many schools adopted distance teaching, so when the pandemic declined, how students could safely attend classes in classrooms and how campuses could be reopened became the greatest focus points for numerous pieces of research and discussions.

2. IAQ is generally worse than outdoor air quality: in some studies, through the monitoring of instruments, researchers found that people worked at home for longer periods of time and that most of the doors and windows were closed to avoid virus infection. In the absence of fresh air, the IAQ is worse than the outdoor air quality.

3. Ventilation is a commonly recommended control strategy: most studies also found that strengthening and improving indoor air flow to reduce the concentration of pollutants does help to improve air quality and to prevent the spread of epidemics. At the same time, among many ventilation strategies, increasing natural ventilation and manually opening windows became the most common suggestions.

To reduce the risk of the COVID-19 infection, the outdoor ventilation rate should be increased to the maximum operational capacity of the indoor ventilation system, which can be two or more times greater than that of the normal operation mode per the existing standard [6]. Practically, mechanical systems or opening doors and windows can both provide adequate ventilation [30,31]. The main advantage of natural ventilation is that it is a theoretically cheap and easy-to-use solution (opening the windows sufficiently), but it also disturbs the thermal regulation of the room [25]. Relatively, the implementation of mechanical ventilation is considered the best strategy to ensure adequate air quality, as it can be a additional, precise adjunct to the control procedures [30,32].

In order to effectively improve IAQ, using air purification and filtration is another noteworthy option to consider [30,33]. When the air outdoors is polluted by particulate matter (PM), filtration is especially important. This is essentially because PM is not a direct cause of COVID-19 infection, however, these pollutants have been shown to be a transporter of bioaerosols.

### 1.2. Clean and Safe Certification

#### 1.2.1. Confidence Indicators for Safe Indoor Spaces

In essence, the “Clean and Safe” marks are designed for epidemic prevention. Since the end of 2019, the COVID-19 pandemic has continued to spread, governments have been forced to lock down big cities, and people have mostly chosen to reduce their participation in outdoor events, which has greatly reduced various economic and tourism activities. In order to gain the trust of the public and allow them to travel confidently, the “Clean and Safe” marks have been specially designed. So far, organizations that have adopted such marks can be roughly divided into three categories: governments, international professional associations, and local professional organizations. Here are some examples to illustrate:

1. Portugal: the “Clean and Safe” stamp was created in 2020 by Turismo de Portugal to recognize companies and leisure activities that comply with the health and safety recommendations and guidelines issued by the National Tourist Authority (NTA) to avoid the risk of contracting COVID-19. It was an action intended as a support tool in crisis management for companies and it assisted the corporations to execute the government’s action plans through free training and provisions. The stamp applied in the three kinds of programs: public health (including COVID-19 and other diseases), extreme phenomena and collective risks, and international constraints [34]. In 2022, the Portuguese government approved a total of 22,164 places to post the certification stamps, trained 42,520 people, and executed 1787 inspections [35].

2. Switzerland: Switzerland Tourism ST mentioned in 2020 that the “Clean and Safe” label was launched to boost visitor confidence through clear and standardized labeling. This label lets travelers know that they are protected by a safe concept established by the Confederation and industry associations, which is mandatory and checked by the cantonal authorities. As the end of 2020, there are 4000 companies from various tour sectors, including hotels, restaurants, conference events, health, public transportation, lake navigation, ropeways, etc., applied for the label and they ordered a total of 15,000 stickers to provide notice to visitors or tourists [36].

3. Malaysia: on July 7th in 2020, the “Clean and Safe Malaysia” certification program launched the first industry-wide hygiene and safety label in the country during the COVID-19 pandemic. The label was initiated by the Malaysian Association of Hotels (MAH) and it was designed for hotels/resorts that comply with local regulatory requirements and international standards. At the same time, MAH has over 1000 hotel members spread across 13 regions and works with the private sector, the government, and institutions such as the Malaysian Ministry of Tourism, Arts and Culture (MOTAC) [37].

4. Serbia: in May 2020, the Business Association of Hotel and Restaurant Industry of Serbia (HORES) created a “Clean and Safe” stamp, which is supposed to provide a certificate for hotels and restaurants marking them as safe facilities for tourist activities. This stamp complies with the guidelines of the WHO and domestic health institutions to prevent the spread of COVID-19 and other infections. By introducing a single, identifiable certification, potential guests can learn about safe areas and book them for themselves and their families [38].

5. World Travel and Tourism Council: the “Safe Travels” stamp is the world’s first global travel and tourism safety and hygiene stamp designed specifically to prevent COVID-19 and similar outbreaks. In particular, the “Safe Travels” stamp is a self-assessment, not a certification. Therefore, countries, destination authorities and companies who apply the stamp must confirm that they have implemented safe travel protocols and will ensure their continued compliance [39]. At present, the stamp is used in six regions including the Americas, Africa, the Asia–Atlantic region, Europe, the Middle East and the Asia–Pacific region, and it is applied by a total of 88 countries and areas.

By reviewing the design and use of the “Clean and Safe” mark, the main purpose of these marks is to gain the trust of tourists and to allow them to travel with confidence. Therefore, most of the indoor places that post such marks are restaurants and tourism-related indoor places.

#### 1.2.2. Application in Taiwan

In 2011, the Taiwanese government passed the “IAQ Act” and announced two sets of specific listed places that must comply with the law, which one set in 2014 and the other set in 2017. These listed places were divided into 16 categories. There are 1614 indoor places regulated and inspected yearly. Taiwan’s Environmental Protection Agency (TW EPA) originally planned to promote a third batch of specific places to expand the scope of the regulation, but there were too many disputes in the planning process which led to the program being suspended for a number of years.

However, in 2021, the TW EPA began to promote self-management IAQ certification which expanded the indoor places to 19 categories and distinguished between the “excellent” and “good” levels. According to the statistical results (published in August 2021), the TW EPA issued 38 marks to public and private indoor places, including 25 excellent levels and 13 good levels, and 31 applications are still pending. For non-specific places, which apply voluntarily, there is a one nursing house that obtained the excellent level mark, and two cases are still going through the application process. At present, the official certification promoted by the TW EPA is still in the initial stages, and the number of places that pass the certification will continue to increase significantly. Besides this, the TW EPA also plans to build an online map by the end of 2022 to make the information more open and transparent. People can check the signs and maps on the Internet and find more high-quality, safe indoor environments.

In addition to the official government certification, there is also a non-profit professional organization in Taiwan that promotes the “Safe and Clean” certification. In 2021, the Taiwan Indoor Environment Quality Management Association (TIEQMA) refers to relevant foreign experience and established a “safe and clean certificate” system and have “Platinum” and “Diamond” as two levels according to the requirements of the inspection standards. It sets strict bacterial safety and cleaning standards, while considering epidemic prevention and IAQ management, and it also assists consumers to create a better public health environment. The association issued platinum level certification to five kindergartens and one special education school in 2021 and it assisted Fullon Hotels and Resorts (Taipei branch) to obtain platinum certification by executing educational training and technical guidance in 2022.

Compared with other empirical studies on IAQ and COVID-19, the similarities between them are that they all explore ways of improving IAQ to reduce the spread of COVID-19 [13,14,23,24,25,26,27,29], however, the biggest difference is the design and choice of research methods. For example, most of the studies have used instrumental monitoring [13,14,21,22,23,24,25,28,29], and a few have used literature reviews [20] or participant observation [26]. Accordingly, this research has the following characteristics: first, this is a first-hand survey of Taiwanese people’s perceptions of epidemic prevention and IAQ, and it also explores the public’s acceptance of the “Clean and Safe” mark; second, the questionnaire design included the background information of the respondents, their opinions on epidemic prevention strategies and IAQ, and at the same time, it inquired about the possibility of establishing “Clean and Safe” marks, and through seven indoor places frequently visited by the public, the research compared their differences; finally, the survey also conducted an in-depth analysis based on the geographic location of the respondents to explore the impact of different actual living environments. Therefore, this research is an innovative and unique empirical arrangement, it has not only combined the issues of epidemic prevention and IAQ, but it has also conducted a difference analysis through seven common indoor places based on the actual basic information of the respondents.

As mentioned above, relevant studies pointed out that various enhanced ventilation strategies can effectively prevent the spread of the COVID-19 pandemic. However, the focus of these studies is mainly on schools and specific workplaces, and there is little discussion and research on areas that the general public often visits. At the same time, if the “Clean and Safe” mark expanded to other places, what are the public’s opinion and acceptance? This is also worth to deeply analyze. Accordingly, the main purpose of this article is to present the epidemic prevention strategies that Taiwanese people often applied during the COVID-19 pandemic, as well as their subjective evaluation of IAQ, and the acceptance of the “Clean and Safe” mark, which needs to be strengthened and improved. Through the comparative analysis of seven indoor places where people often stay, it showed the differences in public perception about IAQ during the COVID-19 pandemic, and the promotional settings of the “Clean and Safe” mark.

## 2. Materials and Methods

### 2.1. Research Design and Methods

In order to understand the public’s opinions on improving IAQ during the COVID-19 pandemic and explore the acceptance of establishing the “Clean and Safe” mark, this research specially designed a questionnaire for further exploration. There are two sets of designs: the first one takes the strategies to improve IAQ during the pandemic as the independent variable, and the IAQ perception of seven indoor places as the dependent variable, and it is used to explore the impact of different improvement strategies at those locations; the second design, also taking the ”Clean and Safe” marks as the independent variables and the IAQ perception of seven indoor places as the dependent variable, was used to explore the promotion on the various places. Therefore, this research proposes the following hypotheses:

**Hypothesis 1 (H1)**. *The IAQ improvement strategies will affect the evaluation of different indoor places*.

**Hypothesis 2 (H2)**. *The promotion of the “Clean and Safe” mark will affect the evaluation of different indoor places*.

In order to ensure that the respondents have a certain representativeness, this research specially conducted an online survey for the whole of the Taiwan region, including the areas with high economic development and a high degree of urbanization (western Taiwan), as well as outlying islands, areas with poor economic development and rural areas (eastern Taiwan). For the feasibility and convenience of the investigation, this research cooperated with TIEQMA, one of the few non-governmental professional organizations in Taiwan that focuses on IAQ-related issues. It also brings together experts in various fields such as environmental protection, air conditioning, construction, and upgrading technology to promote the IAQ industry to formulate unified standards, technical guidance, education and training, and to assist in the promotion of relevant laws and regulations.

Therefore, this survey was conducted with TIEQMA’s annual public welfare activities, which regularly promote IAQ-related information and improvement methods. At the same time, during the investigation period, the association mobilized many volunteers, university professors and experts to randomly invite people who were concerned about IAQ issues to fill in the online questionnaire. Each respondent fully understood the purpose of the questionnaire, and the relevant information is to be used.

During the design process of the questionnaire (see the Appendix A), this research also consulted and communicated with TIEQMA in a timely manner, which means it has a certain degree of expert validity. Before formal execution, the questionnaire was also pre-tested and revised based on the results. Accordingly, the questionnaire was divided into five parts: (1) basic information of the respondents, including five variables such as gender, age, marital status, education, and living area; (2) the practical experience and knowledge scale section, which also has seven questions; (3) the IAQ and COVID-19 prevention scale section, including four questions (Cronbach’s Alpha = 0.726); (4) promotion of the “Safe and Clean” mark scale section—only four questions are required to be answered (Cronbach’s Alpha = 0.870); (5) certification of places scale section, for which there were seven questions that respondents needed to fill out (Cronbach’s Alpha = 0.934).

### 2.2. Data and Survey Background

In 2021, the COVID-19 epidemic in Taiwan begun to spread widely and a level 3 alert was activated on May 15th. The main epidemic prevention measures taken by the public during this period were the wearing of masks and maintaining social distancing. Some people were vaccinated, and schools and workplaces had also begun to adopt distancing methods. This wave of the epidemic gradually declined until August. This research chose to adopt an online survey method, which was conducted between 26 October and 30 October.

In terms of geographical and environmental features, Taiwan is a long and narrow island, including some small outlying islands, with a population of about 23 million. It is roughly divided into two parts: western TW is the main residential area, and the north, middle and south areas each have a metropolitan area. The economic development of this area is good, especially in the north of Taipei City and New Taipei City; in the eastern area, there is a canyon terrain, which is mostly close to the coast. With the a reduced population distribution, the economic development is slow, and the external transportation is inconvenient. Therefore, western Taiwan is the most densely populated area with the best economic development. People’s living and consumption standards are high. Relatively, this area has serious air pollution problems, especially in the metropolitan and industrial parks.

Due to limited funding, this research only used online surveys and convenience sampling, and the results could not be applied to the total Taiwanese population. Finally, in this survey, there were 1206 copies of valid questionnaires, including 1165 copies from the western area and 41 copies from the eastern area.

## 3. Results

### 3.1. Descriptive Analysis of the Results

#### 3.1.1. Features of Samples

According to the basic information of 1206 respondents, the main components are as follows: 96.4% of the respondents lived in the western area of Taiwan, 57% of the sample are female, 31.4% of the respondents are 31 to 40 years old, 54.7% of the respondents are married, and 48.8% have a university education. Furthermore, the samples collected in this research have the following characteristics (see Table 3):

Firstly, the respondents were mostly female, younger, married and highly educated, and the most majority lived in western Taiwan.

Secondly, more than half of the respondents knew the laws related to IAQ, and 96.6% knew that COVID-19 can be transmitted through the air.

These results are not consistent with the real demographic characteristics of Taiwan. The main reason for this bias is that the survey was conducted online and uses a convenience sampling design. Despite the above limitations, these data still have certain referral value.

In the practical experience and knowledge scales, this research designed questions related to understanding the real situation of the respondents. First, 95.6% of the respondents knew that COVID-19 could be prevented by improving IAQ, and 92% knew that a high CO_2_ concentration and poor ventilation would increase the risk of infection. Second, 73% of the respondents were relatively unfamiliar with the other improved IAQ technologies, such as UV technology (see Table 4).

Third, most respondents were aware of the transmission mode of COVID-19, and 92.4% recognized that physical contact, droplets, and air could transmit the virus (see Table 5).

Finally, 92.1% of respondents pointed out that the main epidemic prevention measure taken by the government is the wearing of the masks, and the others are working from home and maintaining social distancing (74.9%). Regarding how to improve IAQ, nearly half of the respondents were aware that the government promoted the strategies (see Table 6).

#### 3.1.2. Description of Scales

Three scales were designed for this research to ask respondents about their perceptions and attitudes towards improving IAQ and the “Clean and Safe” mark, as well as their evaluation of specific indoor places. These scales used a five-point Likert scale (5 = strongly agree), and the results were analyzed using the mean and standard deviation (descriptive statistics) (see Table 7).

**Figure 1:**
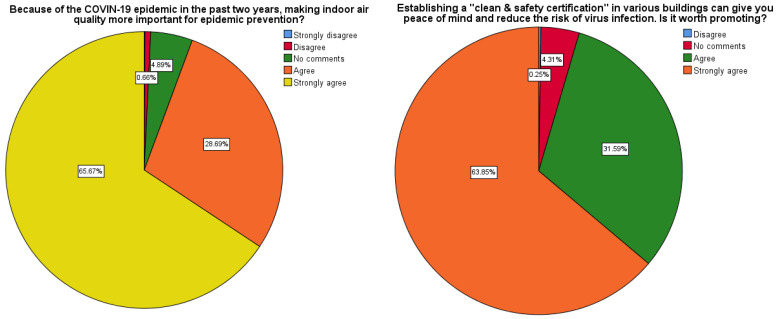
The importance of IAQ and the cognition of “Clean and Safe” certification.

According to the analysis results, 94.4% of the respondents agreed that maintaining IAQ during the pandemic is very important for prevention (strongly disagree is 0.09% and couldn't display properly in Figure 1). In addition, 95.4% of them also pointed out that the “Clean and Safe” mark certification, which contributes to the epidemic prevention effect, should be promoted in public places (see Figure 1). According to Table 7, the following findings can be summarized:

First, among strategies to improve IAQ, ventilation had the highest average (M = 4.6061) and the most concentrated score (smallest standard deviation; STD. = 0.54347), indicating that the respondents agreed that this strategy would improve IAQ the most.

Second, the respondents also mostly agree that the “Clean and Safe” mark can provide confidence and reduce the risk of infection (M = 4.5904; STD. = 0.58590);

Finally, among the seven designated indoor places, long-term care centers are the locations most in need of certification, which also means that the IAQ needs to be improved and strengthened to prevent COVID-19 in those locations (M = 4.5887). This is because most people in long-term care centers are either elderly or frail patients, or injured people who are inconvenient to move. They have been in an indoor environment for a long time without being able to move freely, so they need fresh indoor air to maintain their health.

Overall, the responses in the survey were positive and fully reflected the real situation in Taiwan. In addition, if a specific place got the certification, it increased the willingness of the people to stay and use the facilities. Relatively speaking, the respondents’ opinions on the application of UV/air purifiers to improve IAQ were slightly low and divergent (M = 4.1012; STD. = 0.81156).

### 3.2. Hierarchical Regression Analysis of IAQ, Marks and Indoor Places

The main purpose of using hierarchical regression analysis is to test for any significant differences in the scales using the IAQ and “Clean and Safe” mark promotion as the independent variables, the specific places as the dependent variable, and testing whether there were significant differences among the seven indoor places. This research also used the respondents’ four basic information variables and three practical situations as the control variable: gender (1 = male; 0 = female), age (1 = above 40; 0 = under 39), marital status (1 = married; 0 = unmarried), education (1 = college; 0 = high school), area (1 = east; 0 = west), knowledge of the law (1 = yes; 0 = no), and airborne infection (1 = yes; 0 = no).

#### 3.2.1. Ventilation Is the Key Strategy to Improve IAQ

In the IAQ and COVID-19 prevention scale, this research designed three improvement strategies: a UV/air purifier, ventilation, and regular cleaning. Therefore, we used these strategies as independent variables and tested them by regression analysis to find out whether there was a significant difference for the specific places scale. Based on the results in Table 8 and Table 9, after comparing the seven selected places (*p* < 0.001 for all models; all of the models had an Adj R^2^ between 0.416 and 0.499), it was found that ventilation was the most important factor agreed by the respondents (the B value is the highest and most significant); regular cleaning and disinfection are secondary options, while UV/air purifiers have the lowest values. In other words, respondents generally agreed that enhanced ventilation is the most important strategy to improve IAQ strategies in specific indoor places.

From the analysis results of the control variables, marital status is also an important factor. Married people in particular will pay special attention to IAQ and epidemic prevention measures in designated places (significantly in the elevator, dwelling, office, and kindergarten models). As for other control variables, they vary according to the designated places: for example, young people are concerned about dwellings (β= −0.108 **), men are focused on kindergartens (β = 0.078 *) and long-term care centers (β = 0.061 *), and those who have knowledge of the law are particularly concerned about the conditions in elevators (β = 0.090 **). These results may explain that married people are more concerned about environmental cleanliness; men in Taiwan are often the ones who are responsible for payments, and they care especially about value for money.

#### 3.2.2. Strengthen Promotion and Display Information

In the “Clean and Safe” mark scale, this research also designed three concepts: benefit, promotion and display methods. Again, we used these to test the effect of the scale on specific indoor places. According to the results in Table 10 and Table 11 (*p* < 0.001 for all of the models; all of models had an Adj R^2^ between 0.426 and 0.509), the promotion of marks also strongly affects the public’s evaluation of the locations, and the focused items are also different by the type of locations; the overall regression model has a prediction rate of about 45%. From the category of specific places, the respondents identified that it is necessary to strengthen the promotion of the marks and set them up in elevators (β = 0.280 ***), restaurants (β = 0.336 ***), and offices (β = 0.299 ***). Gyms (β = 0.279 ***), dwellings (β = 0.331 ***), and long-term care centers (β = 0.270 ***) should emphasize the display of the marks at entrances and exits., and kindergartens should ensure safety and reduce the risk of infection (β = 0.274 ***).

In addition, we found that the background information of the respondents affects the evaluation of the indoor locations: women pay more attention to the restaurant environment (β = −0.062 *), young people use the information to refer to selecting houses (β = −0.101 *), those who are married (β = 0.071 *) and those who have an Knowledge of the law (β = 0.074 *) are concerned about elevators, married respondents are concerned about the conditions in the gym environment (β = 0.066 *), and those with a high school educational background care about the environment in kindergartens (β = 0.073 *).

## 4. Discussion

During the COVID-19 pandemic, indoor environments and air quality became very important factors, and they are also closely related to the measurement and assessment of risks [6]. The purpose of this research is to examine the IAQ improvement strategies adopted by various specific indoor places during the COVID-19 pandemic, and the effect reinforced by the safety marks. From the above results, it has been shown that the enhancement of ventilation to improve IAQ is the most preferred choice of respondents (the B value is the highest and most significant), while the establishment of marks also has different effects in indoor places (significant in all of the models).

Looking back to the COVID-19 situation in 2021, compared to other countries, both the number of confirmed cases and deaths increased smoothly in Taiwan. Generally, the TW government’s COVID-19 prevention strategy mainly focuses on the cleanliness of individuals and the indoor environment, such as the use of alcohol for disinfection, and measures to reduce the risk of infection, such as wearing masks, maintaining social distancing, and going to indoor locations and registering with a real name. In fact, vaccination is an important anti-pandemic strategy widely applied around the world, but at that time people did not have immediate access to the vaccine. The main reason is the evident supply shortage which caused a lot of controversy and confusion in the vaccination process. This situation did not improve until September, and people still need to make an appointment to be vaccinated.

Relatively, the TW government has rarely explained how to improve IAQ to enhance the effect of epidemic prevention and establish a confidential indoor environment through the safety mark certification. In fact, prior to this online survey, the Taiwanese government executed a level 3 alert (which ended on 26 July 2021), and the main epidemic prevention strategies adopted by the Center for Disease Control in Taiwan (TW CDC) were: strengthening of handwashing and cleaning, the wearing of masks, closing entertainment locations, allowing food in restaurants to be taken out only, and indoor gatherings being restricted. In other words, with regard to the strategies and techniques used to improve IAQ, the public were often informed by news and media reports. Therefore, increasing ventilation by opening a large number of windows is the most common method chosen by the public, mainly because it is very convenient and low-cost. Moreover, indoor locations, which mostly depend on air conditioners to increase ventilation, usually strengthen the indoor disinfection regime at the same time.

As relevant research points out, IAQ during the COVID-19 pandemic is very likely to be worse than outdoor air quality. However, the TW government only disclosed the indicator values for outdoor air quality and established more than 10,000 monitoring sites. As the pandemic continues, people will frequently stay indoors. Except for the specific places listed by the TW EPA, which regularly detected the IAQ value every six months at least, the IAQ value of other places is not directly displayed. This is the reason why most of the respondents prefer to strongly promote the safety mark, and immediately display the important IAQ values at the entrance and exit of indoor locations to ensure safe travel.

## 5. Conclusions

Thus far, COVID-19 has been spreading for several years, has deeply affected the daily lives of people all over the world, and has also caused many casualties and huge economic losses. At the same time, COVID-19 has indeed led people to reducing how often they go out and it has led to people spending more time in indoor spaces. As recommended by the WHO, when one stays indoors, one should open a window to increase the amount of natural ventilation. The survey conducted in this research found that respondents generally agreed at a rate of 94.4% that improving IAQ through ventilation strategies could prevent the spread of the COVID-19 pandemic. Another finding is the recommendation to strengthen the establishment of clean and safe marks and IAQ information displayed (95.4% agree). These findings also partially verify the two hypotheses proposed in this research (significant and predictive in all of the models), but they cannot be applied to other countries and regions. This research links public perceptions of IAQ and certification marks during the COVID-19 pandemic, so these findings indeed serve as a reference for the business, companies, and governments of Taiwan.

How to maintain IAQ and provide a safe indoor environment are the main challenges in the current spread of COVID-19. From this perspective, looking at the actions taken by the Taiwanese government, the following aspects need to be improved: first, there is little analysis and guidance on strategies and techniques for improving IAQ, especially in enclosed indoor spaces where natural ventilation cannot be applied; second, the TW EPA began to promote IAQ certification in 2021 and adopted a self-management approach to encourage various locations to apply for certification, but the incentives for this system are still insufficient, and the actual number of certifications still needs to be observed; third, IAQ is a dynamic process, which will change with the pollutants in the air, temperature, humidity and other factors. In many indoor places, especially public places where people gather, the IAQ value may change drastically in a short period of time. Therefore, TW government should strengthen the display of indoor air quality information in public places instead of conducting regular testing.

The impact of COVID-19 on people’s daily lives is still present and it has significantly increased the amount of time people spend indoors. As the pandemic changes, the above challenges could become more difficult, and it is also necessary to take effective action. It is undeniable that the COVID-19 pandemic has changed the focus and content of IAQ research, and there are still other related issues that need continuous exploration.

## Figures and Tables

**Table 1:** Control strategies across different scales and estimated risk reduction factors.

		Scales			
Strategies		Person	Cubical	Room	Building
	Source control	Hand washing	Surface disinfection	Hand washing	Surface disinfection
		Wearing of face masks		Wearing of face masks	
	Ventilation	Wearable ventilator	Personal ventilation	Displacement ventilation	Wearable ventilator
		Semi-open partitions	Double or triple ventilation		Semi-open partitions
	Air cleaning	Wearable air purifier Wearing of face masks	Local air cleaner	Room air cleaner	MERV 14 filter HEPA filter MERV 14 filter + UVGI HEPA filter + UVGI

**Table 2:** Findings and recommendations from empirical research on IAQ and COVID-19.

Object	Findings and Recommendations
School [21]	Proper behavior and management to maintain the mechanical ventilation and air conditioning systems are key elements in ensuring IAQ and providing a healthy environment.
School [19]	A room with a central HVAC system responds more quickly to internal sources of contamination than a room with only fan coils.
School [22]	Low ventilation rates in indoor environments increase the likelihood of infection by sharing air with large numbers of susceptible individuals, leading to the spread of infectious diseases, and air exchange promotion can be provided by mechanical ventilation to reduce the risk of airborne transmission.
School [23]	During the COVID-19 pandemic, referring to the requirements of IAQ in international ventilation protocols and guidelines, the recommended carbon dioxide value was found, and the concentration was significantly reduced.
Classroom [24]	The most effective and mixed strategy, that is, to ensure the removal of pollutants, is through continuous cross ventilation. Depending on the purpose and usage of the classroom, different levels of openness help to achieve an adequate air quality.
School [13]	Natural and mixed ventilation modes are effective strategies for maintaining IAQ during cold seasons. Manually operated windows are often provided in school classrooms, which enhance the effects of epidemic prevention.
Nursery Building [25]	The air supply volume of IAQ should be related to the number of people and have nothing to do with the size of the room. When ventilation of the room cannot be achieved, a ventilation device equipped with an air filter must provide purified air at the same time.
Working from home [26]	IAQ in homes during the pandemic was worse than in pre-pandemic offices, and SBS symptoms were more frequent when working from home. Working from home may lead to greater health concerns due to poorer IAQ at home. One of the suggestions for improving IAQ in the home can be achieved through behavioral changes such as opening doors and windows; another method is to provide portable air purifiers with HEPA filters.
Dwellings [27]	Replace or improve the filtration system of your home air conditioning and heating system with independent and efficient ventilation systems and improved natural ventilation mechanisms.
Teleworkers [28]	More interventions are needed to improve their IAQ by controlling the sources of these pollutants and promoting greater ventilation. Air quality inside buildings, both in dwellings and at workplaces, is worse than outside air quality, and workers have also been found to experience values that exceed protection thresholds, in particular, the parameters of CO_2_, CH_2_O, PM_2.5_ and PM_10_ and insufficient thermal comfort.
Kindergartens [14]	It provides good results in terms of IAQ according to ventilation practices related to preventing the spread of COVID-19. Measurements show a 30% improvement in the average daily concentration of indoor carbon dioxide during the COVID-19 pandemic.
Railway coaches [29]	Higher per capita air ventilation rates mean a lower risk of COVID-19 transmission.

**Table 3:** The features of the respondents in the survey.

Items		Frequency	Percentage
Gender	Female Male	668	57%
		518	43%
Age	Under 20 21–30 31–40 41–50 51–60 Above 61	21 221 375 318 194 77	1.7% 18.3% 31.1% 26.4% 16.1% 6.4%
Marital status	Married Unmarried	660 546	54.7% 45.3%
Education	Junior high school High school College University Master’s or PhD	32 232 183 588 171	2.7% 19.2% 15.2% 48.8% 14.2%
Knowledge of the law	Yes No	780 426	64.7% 35.3%
Airborne infection	Yes No	1165 41	96.6% 3.4%
Area	West East	1165 41	96.6% 3.4%

**Table 4:** Knowledge of IAQ and COVID-19.

Items	Improve IAQ to Prevent COVID-19	High CO_2_ and Poor Ventilation Increase the Risk of COVID-19 Infection	UV Technology to Reduce COVID-19
Yes No	1153 (95.6%) 53 (4.4%)	1109 (92%) 97 (8%)	880 (73%) 326 (27%)

**Table 5:** The cognition of COVID-19 infection mode (multiple responses).

Items	Physical Contact	Droplets	Airborne Infection	All of Them
Mode of transmission of COVID-19	75 (6.2%)	85 (7.1%)	41 (3.4%)	1112 (92.4%)

**Table 6:** The cognition of official epidemic prevention measures (multiple responses).

Items	Personal Protection (e.g., Wearing Masks, etc.)	Administrative Controls (e.g., Working from Home and Social Distancing, etc.)	Engineering Controls (e.g., Improved Ventilation and the Use of UV Technology)
Epidemic prevention measures mainly promoted by the TW government	1111 (92.1%)	903 (74.9%)	661 (54.8%)

**Table 7:** The descriptive statistics of scales.

Scale	Items	N	Mean	Standard Deviation
IAQ and COVID-19 prevention scale	The importance of IAQ for epidemic prevention	1206	4.5920	0.62406
	Ventilation	1206	4.6061	0.54347
	UV/air purifier	1206	4.1012	0.81156
	Regular cleaning and disinfection	1206	4.4129	0.66592
“Clean and Safe” mark promotion	Benefits (safety and reduced risk of infection)	1206	4.5904	0.58590
	Promotion setup certification	1206	4.5522	0.60304
	IAQ information displayed at the entrance and exit	1206	4.5713	0.60795
Specific indoor places	Elevator	1206	4.5000	0.66038
	Restaurant	1206	4.5464	0.62247
	Dwelling	1206	4.5141	0.66399
	Office	1206	4.5348	0.62056
	Gym	1206	4.4983	0.64254
	Kindergarten	1206	4.5464	0.62380
	Long-term care center	1206	4.5887	0.58189

**Table 8:** The IAQ and COVID-19 prevention regression (four cases).

Independent Variables	Model 1 Elevator		Model 2 Restaurant		Model 3 Dwelling		Model 4 Office	
	B	(S.E.)	B	(S.E.)	B	(S.E.)	B	(S.E.)
IAQ and COVID-19 prevention								
UV/ air purifier	0.083 ***	(0.019)	0.045 *	(0.018)	0.074 ***	(0.020)	0.078 ***	(0.018)
Ventilation	0.613 ***	(0.030)	0.624 ***	(0.028)	0.579 ***	(0.031)	0.618 ***	(0.028)
Regular cleaning and disinfection	0.201 ***	(0.025)	0.197 ***	(0.023)	0.214 ***	(0.026)	0.172 ***	(0.023)
Control Variables								
Gender (1 = male; 0 = female)	0.038	(0.028)	−0.015	(0.026)	0.014	(0.030)	0.007	(0.026)
Age (1 = above 40; 0 = under 39)	−0.004	(0.031)	0.006	(0.029)	−0.108 **	(0.033)	−0.037	(0.029)
Marital status (1 = married; 0 = unmarried)	0.107 ***	(0.030)	0.069 *	(0.028)	0.087 *	(0.032)	0.064 *	(0.028)
Education (1 = college; 0 = high school)	−0.059	(0.029)	0.002	(0.027)	0.000	(0.031)	0.019	(0.028)
Area (1 = east; 0 = west)	0.022	(0.076)	0.115	(0.071)	−0.007	(0.081)	0.058	(0.071)
Knowledge of the law (1 = yes; 0 = no)	0.090 **	(0.029)	0.014	(0.027)	0.023	(0.031)	−0.004	(0.027)
Airborne infection (1 = yes; 0 = no)	0.074	(0.076)	−0.108	(0.071)	−0.058	(0.081)	−0.085	(0.071)
Constant	0.287	(0.143)	0.673 ***	(0.134)	0.646 ***	(0.152)	0.660 ***	(0.134)
Statistics	N = 1206 F (10,1195) = 116.971 *p* < 0.000 R^2^ = 0.495 Adj R^2^ = 490		N = 1206 F (10,1195) = 120.872 *p* < 0.000 R^2^ = 0.503 Adj R^2^ = 0.499		N = 1206 F (10,1195) = 91.096 *p* < 0.000 R^2^ = 0.433 Adj R^2^ = 0.428		N = 1206 F (10,1195) = 117.596 *p* < 0.000 R^2^ = 0.496 Adj R^2^ = 0.492	

Note: *** *p* < 0.001; ** *p* < 0.01; * *p* <0.05.

**Table 9:** The IAQ and COVID-19 prevention regression (three cases).

Independent Variables	Model 5 Gym		Model 6 Kindergarten		Model 7 Long-Term Care Center	
	B	(S.E.)	B	(S.E.)	B	(S.E.)
IAQ and COVID-19 prevention						
UV/ air purifier	0.069 **	(0.020)	0.051 *	(0.019)	0.033	(0.017)
Ventilation	0.556 ***	(0.031)	0.546 ***	(0.029)	0.565 ***	(0.026)
Regular cleaning and disinfection	0.200 ***	(0.026)	0.208 ***	(0.024)	0.202 ***	(0.022)
Control Variables						
Gender (1 = male; 0 = female)	0.046	(0.029)	0.078 *	(0.028)	0.061 *	(0.025)
Age (1 = above 40; 0 = under 39)	−0.007	(0.032)	0.005	(0.031)	0.002	(0.027)
Marital status (1 = married; 0 = unmarried)	0.048	(0.031)	0.113 ***	(0.030)	0.050	(0.027)
Education (1 = college; 0 = high school)	0.067 *	(0.031)	0.017	(0.029)	0.033	(0.026)
Area (1 = east; 0 = west)	0.078	(0.079)	0.032	(0.075)	0.007	(0.067)
Knowledge of the law (1 = yes; 0 = no)	0.032	(0.030)	0.052	(0.029)	0.021	(0.026)
Airborne infection (1 = yes; 0 = no)	0.046	(0.079)	−0.082	(0.075)	−0.100	(0.068)
Constant	0.620 ***	(0.149)	0.840 ***	(0.142)	0.971 ***	(0.127)
Statistics	N = 1206 F(10,1195) = 86.777 *p* < 0.000 R^2^ = 0.421 Adj R^2^ = 0.416		N = 1206 F(10,1195) = 94.279 *p* < 0.000 R^2^ = 0.441 Adj R^2^ = 0.436		N = 1206 F(10,1195) = 111.735 *p* < 0.000 R^2^ = 0.483 Adj R^2^ = 0.479	

Note: *** *p* < 0.001; ** *p* < 0.01; * *p* < 0.05.

**Table 10:** The “Clean and Safe” mark promotion regression (four cases).

Independent Variables	Model 1 Elevator		Model 2 Restaurant		Model 3 Dwelling		Model 4 Office	
	B	(S.E.)	B	(S.E.)	B	(S.E.)	B	(S.E.)
Clean and Safe Mark promotion								
Benefits (safety and reduced risk of infection)	0.237 ***	(0.044)	0.212 ***	(0.038)	0.268 ***	(0.044)	0.242 ***	(0.040)
Promotion setup certification	0.280 ***	(0.040)	0.336 ***	(0.035)	0.216 ***	(0.041)	0.299 ***	(0.037)
IAQ information displayed at the entrance and exit	0.268 ***	(0.032)	0.284 ***	(0.028)	0.331 ***	(0.032)	0.261 ***	(0.029)
Control Variables								
Gender (1 = male; 0 = female)	−0.003	(0.029)	−0.062 *	(0.026)	−0.034	(0.030)	−0.032	(0.027)
Age (1 = above 40; 0 = under 39)	−0.002	(0.032)	0.008	(0.028)	−0.101 *	(0.033)	−0.038	(0.029)
Marital status (1 = married; 0 = unmarried)	0.071 *	(0.032)	0.022	(0.028)	0.041	(0.032)	0.026	(0.029)
Education (1 = college; 0 = high school)	0-.059	(0.031)	0.011	(0.027)	0.004	(0.031)	0.024	(0.028)
Area (1 = east; 0 = west)	−0.008	(0.080)	0.074	(0.070)	−0.041	(0.081)	0.019	(0.072)
Knowledge of the law (1 = yes; 0 = no)	0.074 *	(0.031)	−0.013	(0.027)	−0.001	(0.031)	−0.024	(0.028)
Airborne infection (1 = yes; 0 = no)	0.106	(0.080)	−0.104	(0.070)	−0.034	(0.081)	−0.065	(0.072)
Constant	0.767 ***	(0.146)	0.857 ***	(0.127)	0.859 ***	(0.147)	0.950 ***	(0.132)
Statistics	N = 1206 F(10,1195) = 90.833 *p* < 0.000 R^2^ = 0.432 Adj R^2^ = 427		N = 1206 F(10,1195) = 126.153 *p* < 0.000 R^2^ = 0.514 Adj R^2^ = 0.509		N = 1206 F(10,1195) = 90.353 *p* < 0.000 R^2^ = 0.431 Adj R^2^ = 0.426		N = 1206 F(10,1195) = 107.819 *p* < 0.000 R^2^ = 0.474 Adj R^2^ = 0.470	

Note: *** *p* < 0.001; * *p* < 0.05.

**Table 11:** The “Clean and Safe” mark promotion regression (three cases).

Independent Variables	Model 5 Gym		Model 6 Kindergarten		Model 7 Long-Term Care Center	
	B	(S.E.)	B	(S.E.)	B	(S.E.)
“Clean and Safe” mark promotion						
Benefits (safety and reduced risk of infection)	0.259 ***	(0.040)	0.274 ***	(0.042)	0.262 ***	(0.037)
Promotion setup certification	0.249 ***	(0.037)	0.269 ***	(0.039)	0.221 ***	(0.034)
IAQ information displayed at the entrance and exit	0.279 ***	(0.029)	0.266 ***	(0.031)	0.270 ***	(0.027)
Control Variables						
Gender (1 = male; 0 = female)	0.032	(0.027)	0.002	(0.028)	0.015	(0.025)
Age (1 = above 40; 0 = under 39)	0.010	(0.030)	−0.003	(0.031)	0.006	(0.027)
Marital status (1 = married; 0 = unmarried)	0.066 *	(0.029)	0.001	(0.030)	0.006	(0.027)
Education (1 = college; 0 = high school)	0.023	(0.028)	0.073 *	(0.030)	0.039	(0.026)
Area (1 = east; 0 = west)	−0.007	(0.073)	0.035	(0.077)	−0.025	(0.068)
Knowledge of the law (1 = yes; 0 = no)	0.025	(0.028)	0.006	(0.029)	−0.006	(0.026)
Airborne infection (1 = yes; 0 = no)	−0.075	(0.073)	0.055	(0.076)	−0.094	(0.068)
Constant	0.933 ***	(0.133)	0.694 ***	(0.139)	1.201 ***	(0.124)
Statistics	N = 1206 F(10,1195) = 105.182 *p* < 0.000 R^2^ = 0.468 Adj R^2^ = 464.		N = 1206 F(10,1195) = 99.037 *p* < 0.000 R^2^ = 0.453 Adj R^2^ = 0.4449		N = 1206 F(10,1195) = 107.493 *p* < 0.000 R^2^ = 0.474 Adj R^2^ = 0.469	

Note: *** *p* < 0.001; * *p* < 0.05.

## Data Availability

Not applicable.

## Appendix A. The Questionnaire of Improving IAQ and Certification to Strengthen COVID-19 Prevention

**1. Basic Information**

1.1 Gender □ Male □ Female

1.2 Age □ < 20 years old □ 21–30 years old □ 31–40 years old □ 41–50 years old

□ 51–60 years old □ 61 years old and above.

1.3 Marital status □ Married □ Unmarried

1.4 Education □Junior high school □High school □College □University □Master’s degree or PhD

1.5 Living area □Western Taiwan □Eastern Taiwan

**2. The Practical Experience and Knowledge Scale Section**

2.1 Do you know that the government has formulated the “Indoor Air Quality Act “?

□Yes □No

2.2 Do you know that COVID-19 can be transmitted through the air? □Yes □No

2.3 Do you know that improving indoor air quality will help reduce the chance of infection with COVID-19? □Yes □No

2.4 Does a too high level of indoor air carbon dioxide represent poor ventilation and increase the risk of COVID-19 infection? □Yes □No

2.5 Do you know that UV technology kills bacteria and is also effective at killing COVID-19? □Yes □No

2.6 Do you know how COVID-19 is spread? (multiple responses)

□Physical contact □Droplets □Airborne infection□ All of them

2.7 What is the main anti-epidemic strategy applied by the government? (multiple responses)

□Personal protection (e.g., wearing masks, etc.)

□Administrative controls (e.g., working from home and social distancing, etc.)

□Engineering controls (e.g., improved ventilation and use of UV technology)

**3. The IAQ and COVID-19 Prevention Scale Section**

3.1 Do you agree that the COVID-19 pandemic that has spread in the past two years has made indoor air quality more important for epidemic prevention? (1 to 5, 5 = strongly agree)

3.2 In the fight against the COVID-19 pandemic, do you think that strengthening ventilation in public places is an important epidemic prevention strategy? (1 to 5, 5 = strongly agree)

3.3 Indoor activity spaces (such as homes or offices) need to be regularly cleaned and disinfected to prevent COVID-19 infection? (1 to 5, 5 = strongly agree)

3.4 Do you think using UV technology or air purifiers indoors is helpful for epidemic prevention? (1 to 5, 5 = strongly agree)

**4. The “Clean and Safe” Mark Promotion Scale Section**

4.1 The “Clean and Safe” mark is worth promoting? (1 to 5, 5 = strongly agree)

4.2 Establishing “Clean and Safe” certification in various buildings can give you peace of mind and reduce the risk of virus infection? (1 to 5, 5 = strongly agree)

4.3 The establishment of “Clean and Safe” certification in different public places should be strengthened? (1 to 5, 5 = strongly agree)

4.4 In order to obtain the “Safety and Clean” mark certification, it is necessary to install indoor air monitoring and display devices at important entrances and exits? (1 to 5, 5 = strongly agree)

**5. The Certification of Places Scale Section**

5.1 During the epidemic prevention period, do you think building elevators in public places also need to improve IAQ and “Clean and Safe” certification? (1 to 5, 5 = strongly agree)

5.2 Would you prefer to dine at restaurants with the “Clean and Safe” mark? (1 to 5, 5 = strongly agree)

5.3 Would you prefer to buy or live in a “Clean and Safe” certified building? (1 to 5, 5 = strongly agree)

5.4 Would you feel more secure at work if the office had a “Clean and Safe” mark? (1 to 5, 5 = strongly agree)

5.5 Would you prefer to choose a gym with the Clean and Safe” mark? (1 to 5, 5 = strongly agree)

5.6 Would you prefer to choose a kindergarten with the “Clean and Safe” mark for your child to attend? (1 to 5, 5 = strongly agree)

5.7 Would you prefer to choose a long-term care institution with the “Clean and Safe” mark? (1 to 5, 5 = strongly agree)
